# Oral complaints in patients with acute myeloid leukemia treated with allogeneic hematopoietic stem cell transplantation

**DOI:** 10.4317/medoral.24647

**Published:** 2021-06-20

**Authors:** Aleksandra Wysocka-Słowik, Lidia Gil, Zuzanna Ślebioda, Barbara Dorocka-Bobkowska

**Affiliations:** 1 Department of Gerodontology and Oral Pathology, Poznan University of Medical Sciences, Poland; 2 Department of Hematology and Bone Marrow Transplantation, Poznan University of Medical Sciences, Poland; 3 ORCID: 0000-0002-5482-3964. Department of Gerodontology and Oral Pathology, Poznan University of Medical Sciences, Poland; 4 ORCID: 0000-0003-3659-7761. Department of Gerodontology and Oral Pathology, Poznan University of Medical Sciences, Poland

## Abstract

**Background:**

Acute myeloid leukemia belongs to proliferative diseases of the hematopoietic system. It is currently the leading indication for allogeneic hematopoietic stem cell transplantation. This study was designed to determine the most common subjective oral mucosa complaints in patients with acute myeloid leukemia after allogeneic hematopoietic cell transplantation, in relation to the type of conditioning used.

**Material and Methods:**

Eighty patients diagnosed with acute myeloid leukemia were assigned to two groups depending on the intensity of the conditioning regimen before transplantation: myeloablative and reduced-intensity chemotherapy. The oral symptoms were evaluated based on an authorial questionnaire designed for this analysis. The following oral mucosa subjective complaints were included: pain, paraesthesia, burning mouth sensation, taste disorders, excessive salivation, halitosis, and dryness of the oral mucosa.

**Results:**

The most commonly reported subjective oral complaint in the examined patients was xerostomia, which was found in 92% of patients during the second visit, followed by spontaneous pain in the mouth (55%), burning (36%), and dysgeusia (20%). It occurred significantly more frequently in patients who underwent myeloablative conditioning. Moreover, it was observed that the frequency of complaints increased considerably after the transplantation, reaching a peak intensity during the second week following the procedure.

**Conclusions:**

Oral complaints significantly decrease the patients' quality of life during the transplantation and may lead to premature termination of the treatment. As the number of transplantations in patients with acute myeloid leukemia increases, further investigations of oral complaints and symptoms induced by the disease itself and by the therapeutic approaches are required.

** Key words:**Acute myeloid leukemia, oral pathology, xerostomia, myeloablative conditioning, reduced-intensity conditioning.

## Introduction

Acute myeloid leukemia (AML) belongs to a heterogeneous group of proliferative diseases of the hematopoietic system. The disease causes uncontrolled clonal proliferation of neoplastic hematopoietic precursors and disturbed production of normal blood cells in blood, bone marrow, and other tissues. AML accounts for about 80% of all acute leukemia cases in adults. The average age of patients is 69 years, and disease risk increases with age (1-3). The treatment of AML depends primarily on the patient's age, general health, cytogenetic, and molecular risk. Treatment with conventional chemotherapy has resulted in total remission in 60-80% of adults with AML de novo under 60 years of age (4,5). AML is currently the leading indication for allogeneic hematopoietic stem cell transplantation (allo-HSCT).

Prior to transplantation, high doses of antiproliferative and cytostatic drugs are administered to the patient (myeloablative conditioning; MAC). The intensive conditioning regimen decreases the risk of relapse after transplantation; however, it is characterized by high toxicity (6). An alternative option of pre-transplant treatment is reduced-intensity conditioning (RIC). It is designed to suppress the patient's immune system enough to accept the donor stem cells while being less toxic than MAC. However, the risk of transplant rejection is higher for this type of procedure. Nevertheless, RIC has extended the application of allo-HSCT to include patients who otherwise would not be eligible candidates for standard conditioning because of their advanced age or comorbidities (6).

Despite the significant progress in the treatment of AML through the application of allo-HSCT, it still results in many complications. High doses of cytostatic drugs induce cytopenia, which increases the risk of viral, fungal, and bacterial infections and leads to the development of gastrointestinal ulceration, often presented as severe oral mucositis (7,8). The use of anti-emetics, anti-depressants, sedatives, and antibiotics, together with chronic stress, may impair saliva production and cause changes to its composition, resulting in the dysregulation of physiological flora of the oral cavity (9,10). Oral symptoms arising from these complications significantly degrade the patients' quality of life after transplantation (11). Because the reduced-intensity conditioning enables the treatment of older adults and patients with coexisting systemic diseases, the number of allo-HSCT procedures is progressively increasing. Therefore there is a need for a thorough analysis of the health status of patients with AML after allo-HSCT, which should include oral health and subjective complaints.

This study was designed to determine the most common subjective oral mucosa complaints in patients with AML after allo-HSCT treatment, in relation to the type of conditioning used.

## Material and Methods

The study group consisted of 80 patients (42 women and 38 men), aged 19 to 69 years (mean 46.6±13.6), diagnosed with AML. All patients underwent allogeneic hematopoietic cell transplantation from December 2015 to July 2018.

Depending on the conditioning, the patients were assigned to one of two groups. The first group consisted of 54 patients (30 women and 24 men), with a mean age of 42.3±11.9 years, who underwent myeloablation therapy (MAC). The other group consisted of 26 patients (12 women and 14 men) with a mean age of 55.5±12.9 years, who were treated with reduced-intensity therapy (RIC). Each patient was examined three times by the same person, an experienced dentist, qualified in the field of oral pathology. The examination was performed with a standardized dental set in artificial light, according to the following scheme used in the Department of Hematology and Bone Marrow Transplantation UMP:

A) preliminary examination in the period preceding bone marrow transplantation from day- 10 to day-7

B) first examination- after transplantation of hematopoietic cells from day +3 to day +7

C) the second examination- after hematopoietic cell transplantation from day +8 to day +14.

The oral symptoms were evaluated based on an authorial questionnaire specifically designed for this analysis. The following oral mucosa-related subjective complaints were included: pain, paresthesia, burning mouth sensation, taste disorders, excessive salivation, halitosis, and dryness of the oral mucosa. Oral dryness was investigated on a three-stage scale, which consisted of: none [0], mild [1], moderate [2], and severe [3] grade of the symptom (12).

The results were statistically analyzed with Statistica.PL ver. 13.0 (StatSoft, Inc., 2014) for Windows with test of the difference between tests with *p*<0.05 considered as a significance level.

## Results

**Figure 1:**
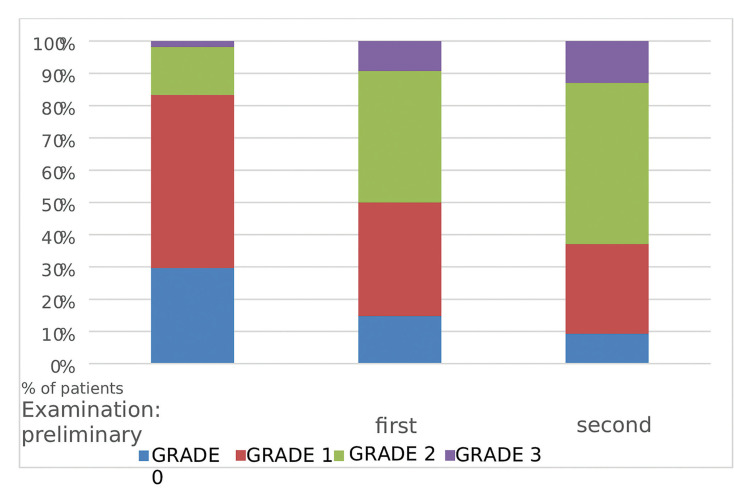
The feeling of dryness in the entire study population after the transplant procedure.

**Figure 2:**
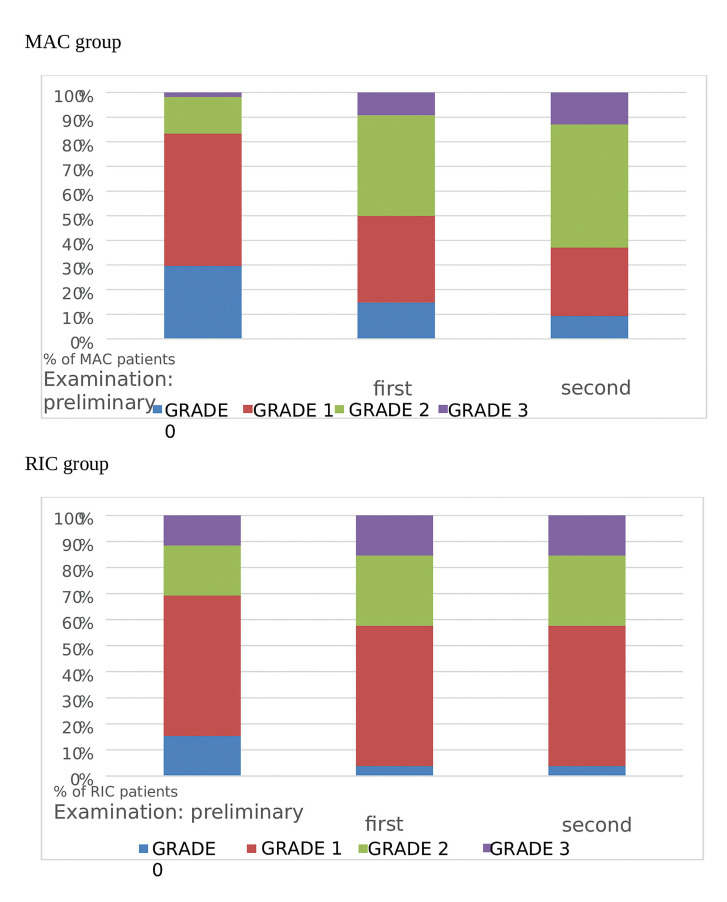
The feeling of dryness in the MAC and RIC groups after the transplant procedure.

The most frequently reported oral complaint was dryness, which occurred in 60 (75%) patients in the preliminary examination. In the post-transplant examinations, this sensation was significantly more frequent and amounted to 71 patients (89%) in the first week (*p*= 0.0212) and 74 (92%) patients in the second follow up (*p*= 0.0038) (Fig. 1). With respect to the type of conditioning considered, each examination showed a progressively higher percentage of patients suffering from dryness in the RIC group compared to MAC (75% vs. 71% in the preliminary examination, 96% vs. 75% in the first week, and 96% vs. 91% in the second examination). However, these differences were not statistically significant. In both groups, the number of patients with a history of dryness increased with each examination. However, statistically significant differences (*p*= 0.0081) were found only in the MAC group only between the preliminary and the second-week examinations (71% vs. 91%) (Fig. 2). Grade 1 (mild dryness) was reported by 43 (54%), 33 (42%), and 29 (36%) patients in the preliminary and two subsequent post-transplant examinations, respectively. The difference was statistically significant between the preliminary and the second examination (*p*= 0.0221). Depending on the type of conditioning, during the preliminary examination Grade 1 dryness occurred in 54% of patients in both groups (29 patients in the MAC and 14 in the RIC group). However, in the RIC group, it remained at the same level during the subsequent post-transplant follow-ups, while in the MAC group a significant reduction was observed (35% and 28% in the two subsequent examinations; *p*= 0.0470 and *p*= 0.0060, respectively). The difference between the percentage of patients with Grade 1 dryness in the MAC group (28%) and in the RIC group (54%) in the second week was statistically significant (*p*= 0.0236) (Fig. 2). Moderate dryness (Grade 2) was most frequently reported by patients in the MAC group (22 patients, (41%) in the first week and 27 patients (50%) in the second week after transplantation). In both post-transplant examinations of the RIC group, Grade 2 dryness was reported by 7 patients (27%). The proportion of patients with a history of Grade 2 dryness in the preliminary examination was similar and reached 15% (8 patients) in the MAC group and 19% (5 patients in the RIC group. Statistically significant differences were found between the patients with moderate dryness examined during the preliminary examination (16%) and at the first follow-up (36%) (*p*= 0.0039) and between the preliminary examination and the second follow-up (42%) (*p*= 0.0003) (Fig. 1). Similarly, the MAC patients were significantly more likely to complain of Grade 2 dryness during the first and second examinations compared to the preliminary examination (*p*= 0.0026 and *p*= 0.0001). Severe dryness (Grade 3) was reported by a total of 9 (11%) patients during the first examination and by 11 (14%) patients in the second post-transplant examination. Considering the incidence of Grade 3 dryness and the type of conditioning, no statistically significant differences were found between the two groups in subsequent examinations. During the preliminary examination this was reported by 1 MAC patient (2%) and 3 RIC patients (12%), in the first week following transplantation- by 5 MAC patients (9%) and 4 RIC patients (15%) and in the second post-transplantation week- by 7 MAC patients (13%) and 4 RIC patients (15%). However, it was found that in the MAC group, Grade 3 dryness was significantly more frequently reported in the second week compared to the preliminary examination (13% and 2%, respectively; *p*= 0.03).

**Figure 3:**
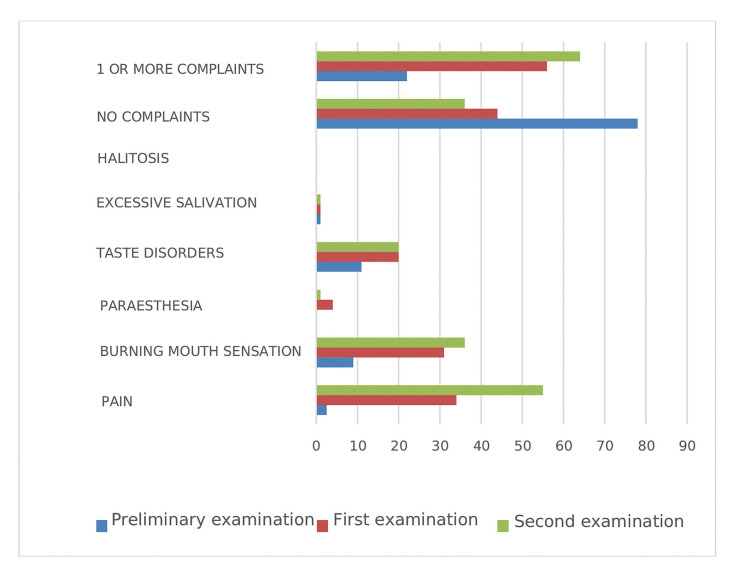
Symptoms presented after the transplant procedure by patients with AML who underwent allo-HSCT.

The second most frequently observed complaint was a spontaneous pain in the mouth, which was reported by more than half of the patients: 47 people (55%) in the second week following transplantation and 27 patients (34%) in the first week after transplantation (Fig. 3). In the MAC group, the pain was experienced by 20 patients (37%) in the first week and by 35 (65%) patients in the second week following transplantation, while during the preliminary examination, it was reported by only one patient, (2%). Patients in the RIC group reported this condition less frequently compared to the MAC group: 1 patient (4%) in the preliminary examination, 7 (27%), and 9 (35%) in the first and second post-transplant examinations, respectively. The difference between the percentage of patients experiencing the pain in the second week in the MAC and RIC groups (65% and 35%) was statistically significant (*p*= 0.0115).

The burning sensation of the oral mucosa was reported by a total of 25 (31%) patients during the first examination and by 29 (36%) during the second examination (Fig. 3). Prior to transplantation, this complaint occurred in 7 (9%) patients, including 4 (7%) in the MAC group and 3 (12%) in the RIC group. In the first week after transplantation, the burning sensation was reported by 20 (37%) patients in the MAC group and 5 (19%) in the RIC group. Both in the preliminary and in the first examination, the two differences were not statistically significant. During the second examination, the incidence of burning sensation increased to 46% (25 patients) in the MAC group, while in the RIC group, it decreased to 15% (4 patients), which was statistically significant, *p*= 0.0068.

Taste disturbance (dysgeusia) was reported relatively frequently. A total of 9 (11%) patients had a history of such a disturbance before the commencement of the study and 16 patients (20%) reported this in both post-transplant examinations (Fig. 3). In the MAC group, 7 patients (13%) complained of dysgeusia, and in the RIC group, 2 patients (8%) reported this complaint previously during the preliminary examination. In the first and second week after transplantation, dysgeusia was reported by 10 (19%) and 11 (20%) patients in the MAC group and 6 (23%) and 5 (19%) patients in the RIC group, respectively (all differences were statistically insignificant).

Oral paraesthesia was reported by only 3 MAC group patients (4%) in the first week following transplantation and by one (1%) during the second follow-up. There were no reports of paraesthesia in the preliminary examination (Table 1).

Hypersalivation and halitosis were the least reported symptoms. The former was reported by one patient in the MAC group during both examinations after transplantation and by one patient in the RIC group before the transplantation. No patient complained of halitosis, either before or after transplantation (Table 1).

**Table 1:**
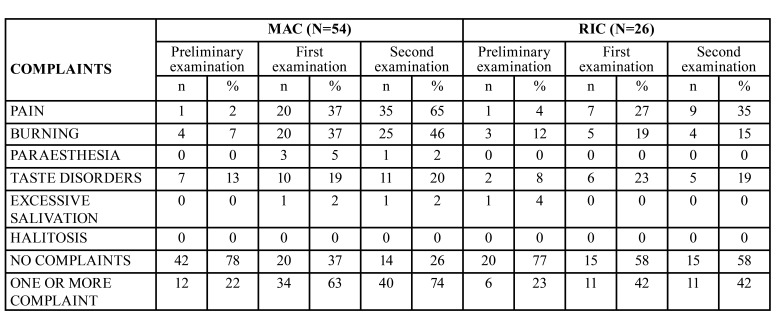
Symptoms of patients from the MAC and RIC groups following the transplant procedure.

**Figure 4:**
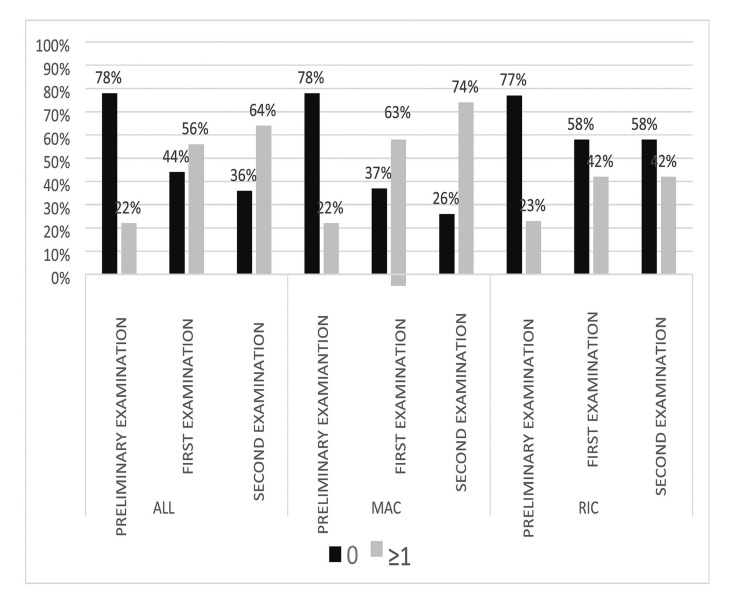
Frequency of the oral symptoms (excluding dryness) compared to the type of conditioning (MAC vs. RIC).

We also assessed the differences between the percentage of patients in the MAC and RIC groups who presented with at least one complaint from those mentioned above (excluding dryness) or did not report any complaints either before or after the transplant procedure (Fig. 4). During the preliminary examination, 62 of the respondents (78%) did not report any symptoms in their history (42 patients or 78% in the MAC group, 20 patients or 77% in the RIC group). During the first week, an increase in the incidence of at least one complaint was observed from 22% (18 patients) to 56% (45 patients) within the entire study population. The highest percentage of patients (40 patients or 74%) complaining of various symptoms was recorded in the second week in the MAC group. In the RIC group, there were equal numbers of complaints during the first and second examination (11 people, 42%). It was found that amongst the patients reporting various symptoms after transplantation, MAC patients accounted for a greater proportion compared to RIC patients (63% vs. 42% in the first study, 74% vs. 42% in the second week). The difference between the groups in the second examination was statistically significant (*p*= 0.0053).

## Discussion

A variety of oral symptoms occur in patients with leukemia seeking initial medical advice (13,14). Oral symptoms reported by the patients involved in this study included: xerostomia, spontaneous pain in the oral region, burning sensation of the oral mucosa, taste disturbances, paraesthesia, hypersalivation, and halitosis.

Based on the previously published reports (15,16), and our study results, the most common oral complaint in post-allo-HSCT patients was xerostomia. Saliva participates in several antimicrobial and protective actions in the oral environment, mainly due to the presence of immunoglobulins, defensins, cystatins, lactoferrin, and buffers (16,17). The secretory action of salivary glands in adults is modified by numerous physiologic factors, including body hydration, weight, time of day, season, gender, body position, size of secretory glands, and psychological stimuli, or stress. These factors determine both the amount and the quality of saliva produced. It is also subject to viscosity, protein and ion content modification (16). Several drugs used during the transplantation procedures may induce xerostomia- the subjective symptom of dry mouth and may interfere with the salivation. These include cytostatics, anticholinergic drugs, anti-depressants, and diuretics (16,18,19). It is not clear how much xerostomia in HSCT patients is accompanied or can be ascribed to medication-induced salivary gland hypofunction and reduced saliva secretion. The salivary reflex, which consists of peripheral and central components, may be interrupted by several drugs and medication-induced salivary gland dysfunction (MISGD) characterized by reduced saliva production (20). The cholinergic muscarinic (M3) receptors of salivary gland acinar cells are the principal peripheral targets for those drugs. Similarly, some of the agents, e.g., tricyclic anti-depressants block muscarinic receptors in the periphery and simultaneously have targets in the central nervous system (20).

In our study group, the frequency and the intensity of this complaint increased in the subsequent evaluations, reaching a peak level in the second week after the transplantation. This could have been due to the increasing impact of cytotoxic drugs on the oral mucosa and from the significant impairment of the immune system efficacy that occurred in patients at this period. In the initial stage of our study, xerostomia was reported by 60 patients (75%), but in the majority of cases, it was considered to be insignificant and could potentially be considered as a drug side-effect (e.g., from anti-depressants and diuretics). The frequency of xerostomia increased in the following weeks, reaching 89% and 92% in the first and second-week post-transplantation, respectively. In contrast to our study results, according to Darczuk, xerostomia appeared in only 33.3% of post-allogenic stem cell transplant patients during the first week after the procedure and in 25% of patients in the third week after the treatment. In the initial phase of her study, only one case of xerostomia was reported. It should be emphasized, however, that this author studied a heterogeneous group of patients with several types of progressive, malignant neoplasms of the blood-forming organs, including acute lymphoblastic and myeloblastic leukemias, chronic myeloid leukemia, diffuse large B-cell lymphoma, Hodgkin's disease, myeloma multiplex, and non-Hodgkin's lymphoma (21). The majority of the literature reports refer to further stages of the post-transplant procedure and combine the oral symptoms with a graft-versus-host disease (GvHD) (18,22). According to Bogusławska-Kapała, the subjective complaint of dry mouth was reported by 95% of patients examined between 3.5 and 10 months after the allogeneic stem cell transplantation (23). Meanwhile, Nakamura *et al*. reported xerostomia in 56% of patients who underwent allo-HSCT in the period from 3 to 34 months (24).

There are only a limited number of studies which have analyzed the influence of the conditioning type on oral complaints and symptoms in AML. The results of our studies indicate that the type of conditioning employed interfered with the dryness sensation in the mouth in AML patients treated with allo-HSCT. Xerostomia was more severe in patients who underwent MAC than RIC, although the difference was not statistically significant. In the MAC group, the frequency and the intensity of mouth dryness increased with every post-transplant examination. However, the number of RIC patients who reported xerostomia before and after the transplant procedure increased by an insignificant number. Our observations remain consistent with the findings of Andersson *et al*., who evaluated the quality of life in patients after allogeneic bone marrow transplants with respect to the type of conditioning (MAC and RIC) over a period of 12 months. In the subsequent examinations, the number of patients reporting dry mouth decreased in the RIC group while remaining at a constant, high level in the MAC group (25).

A high frequency of patients who complained of dry mouth in our study during the first weeks post-transplantation suggests a need to popularize an algorithm of dental procedures in order to reduce xerostomia in patients being prepared for this treatment and those already undergoing this type of therapy.

Spontaneous pain in the oral cavity was the next most frequently observed complaint in our study. It was reported by more than 50% of patients two weeks after transplantation. Moreover, MAC patients reported this complaint more frequently than RIC patients. The burning sensation observed in over 30% of all examined patients in the second week after the transplantation affected more patients after MAC than after RIC conditioning. In the Darczuk study, which examined post-allo-HSCT patients suffering several types of malignant neoplasms of the blood-forming organs, the most commonly reported oral complaints included: spontaneous pain, taste disturbances, and oral burning sensation. As in our study, the highest rate of spontaneous oral pain appeared in the second week after transplantation (21). It was reported by over 80% of patients. In the Andersson *et al*. study, the most common oral symptoms in patients who underwent MAC and RIC for several types of malignant neoplasms in one month after the transplantation were: pain, loss of appetite, and taste disturbances. However, the MAC group reported those complaints much more frequently than the RIC patients. Moreover, the patients on the reduced-intensity conditioning regimen regained a satisfactory quality of life faster than the group on myeloablative therapy (26). Spontaneous pain and burning sensation corresponded to the exacerbation of inflammatory mucosal lesions, which often developed at the early stage of anti-cancer treatment. It could be considered as the adverse effects of cytostatics used during the pre-transplantation period. Antineoplastic drugs, which predominantly aim to eliminate malignant cells, do not act selectively. They also affect other cells with high mitotic rates, including oral epithelium, and odontogenic cells, leading to a disturbed proliferation, maturation, and regeneration (27,28). The intensity of side effects induced by chemotherapy and conditioning regimens for HSCT depends on the type and dosage and the patient's age at the beginning of treatment (27). The oral mucosal barrier injury develops as a consequence of the direct action of cytostatic agentson the oral epithelium and impaired immune response and enhanced activity of free radicals, generated in the presence of cytostatic drugs (29).

Another common oral complaint observed in our study was dysgeusia, reported by 16 (20%) patients in subsequent examinations after the transplantation. According to Epstein, dysgeusia is one of the most commonly reported cancer symptoms (30). Several mechanisms are involved in impaired taste sensation in HSCT patients. First of all, the quantity and quality of saliva interferes with the taste function. Saliva is responsible for food-coating, mastication, and food particle delivery to the taste receptors. It provides oral comfort, and it is required for proper swallowing and speech. Therefore patients who experience hyposalivation may suffer from a disturbed taste function (18,19,30). Secondly, the increased rate of oropharyngeal mucosal infections (e.g., candidiasis) and increased plaque levels commonly observed in patients during the HSCT procedure may affect the taste sensation (7,18,19,30). However, the leading cause of dysgeusia in oncology care is cytotoxicity and neurotoxicity of systemic medications (19). Taste disturbances can be induced by chemotherapeutic agents and their metabolites secreted into saliva and gingival crevice fluid by directly damaging the taste receptors. Taste disorders during chemotherapy typically occur together with mucosal injury, which suggests damage to taste receptor cells in the epithelial taste buds. When a taste change persists, it may reflect decreased turnover rates of taste receptor cells, lack of connectivity between receptor cells and neurons, and possible neuronal damage. Recovery of damaged or lost taste buds may become disturbed over time in those patients when taste progenitor cells are lost (19).

## Conclusions

The results of our study show that xerostomia was the most commonly reported subjective complaint in the oral cavity of patients with AML treated with allo-HSCT. This complaint occurred significantly more frequently in patients who underwent MAC therapy. Moreover, it was observed that the frequency of subjective complaints increased considerably after the transplantation, reaching a peak intensity during the second week following the procedure.

These effects significantly decrease the patients' quality of life during the transplantation and may prematurely terminate the treatment. Considering the continuing growth in the number of performed transplantations in AML patients, further investigations in of oral complaints and symptoms induced by the disease itself and by the therapeutic approaches are required.
